# The Development of the Dance Smart Intervention: A Céilí Dance Exercise Intervention for People Living With Dementia and Their Care Partners (Dance Smart Intervention)

**DOI:** 10.1177/14713012261435732

**Published:** 2026-03-19

**Authors:** Niamh Kelly, Fiona Skelly, Kieran Dowd, Desmond O’Neill, Noel McCaffery, Clare McDermott

**Affiliations:** 1 Department of Sport and Health Sciences, Technological University of the Shannon, Athlone, Ireland; 2 Age Related Healthcare, Tallaght University Hospital, Dublin, Ireland; 3 8809 Centre for Ageing, Neurosciences and the Humanities, Trinity College Dublin, Dublin, Ireland; 4 ExWell Medical, Dublin, Ireland

**Keywords:** physical activity, exercise, dance, intervention development, care partners, people living with dementia, mixed-methods

## Abstract

Dance is a form of exercise that engages motor, cognitive, social, and emotional skills (Charras et al., 2020). Céilí dance is a traditional Irish social dance offering physical and psychological health benefits, with the added benefit of a social element. People living with dementia and care partners have low levels of physical activity (PA) despite understanding the range of benefits (van Alphen et al., 2016). This study aimed to develop a person-centred dance - based exercise intervention for people living with dementia and care partners. The Medical Research Council Framework (MRCF) and behaviour change wheel (BCW) informed the intervention design. A literature review, focus groups and interviews with people living with dementia and care partners were completed to explore experiences and preferences of physical activity and exercise. Findings were mapped to intervention functions and further developed into intervention components using selected behaviour change techniques (BCTs). A stakeholder group and a participant advisory group (PAG) reviewed and refined the intervention. The intervention targeted increasing physical activity and exercise (PAE) in people living with dementia and care partners. Thirteen statements were generated from nine studies. Twenty-six participants, (nine people living with dementia, seventeen care partners) engaged in focus groups or interviews. Analysis identified physical and psychological capability, physical and social opportunity, as the key constructs for change. These aligned with six out of nine intervention functions, leading to the identification of twenty-one appropriate BCTs. These were reformed into six intervention components. These included: a supervised dance exercise class, an induction session, assessments of physical and psychological health, a goal setting and action planning session, daily prompts for class, and a social aspect. This Céilí dance exercise intervention is a theory-informed, systematically designed intervention tailored for people living with dementia and care partners. It has the potential to improve PAE in this population. Future research should assess its feasibility and effectiveness.

## Introduction

Dementia is a neurodegenerative syndrome characterized by the loss of two or more cognitive functions e.g. judgement, thinking, memory (Arvanitakis et al., 2019). Age is the greatest risk factor for developing dementia, with incidence increasing after the age of 65 (Guerreiro & Bras, 2015). As the global population ages, dementia prevalence is rising with over 57 million cases of dementia across the world and there is currently no cure (Livingston et al., 2024). This is predicted to increase to over 150 million by 2050 (Nichols et al., 2022). An estimated 64,000 people in Ireland are living with dementia and 90% of these individuals are over 65 (Nichols et al., 2022; O’Shea et al., 2019; Rogan et al., 2023).

The World Health Organisation (WHO) has highlighted that more research for care and treatment of dementia is needed to assist in the overall management of the disease (World Health Organization, 2017). Due to the limitations of pharmacological treatments, non-pharmacological lifestyle-based interventions are increasingly explored. They have identified a positive impact on physical function, behavioural, and emotional symptoms (Park & Cohen, 2019). However, this research is currently limited, due to low recruitment rates, and long research development times (Pickett et al., 2018). Both pharmacological and non-pharmacological approaches can help slow the decline of functional abilities including balance, reduce fall rates and support mental health such as reducing anxiety (Nimmons et al., 2024; Park & Cohen, 2019). Interventions specific to people living with dementia and care partners are required to allow us to understand improvements needed in current care practices.

Various intervention types are beneficial for people living with dementia, including art, such as painting or drawing sessions, music, including engaging and passive music sessions, behavioural therapy, validation therapy and physical activity and exercise (PAE) (Douglas et al., 2004). Physical and psychological health can improve following general exercise in people living with dementia (Cunningham et al., 2020). Maintaining physical function helps older adults preserve their independence and community living (Tornero-Quiñones et al., 2020). Physical activity and exercise improves mobility, strength and mental wellbeing, including reduced depression (Aartolahti et al., 2020; Fox et al., 2007; Gothe et al., 2020; Lewis et al., 2020; Nimmons et al., 2024). Physical activity and exercise interventions should be adaptable and allow for the inclusion of diverse populations, such as people living with dementia and care partners. Despite the numerous health benefits, many people living with dementia and care partners do not participate in adequate levels of PAE per week (Boyle et al., 2015). The WHO recommends 150–300 minutes of moderate - vigorous PAE weekly for all older adults, including those with a chronic illness (WHO Guidelines on Physical Activity and Sedentary Behaviour, 2024). The PAE should include multicomponent activities, such as balance, strength and aerobic exercise (WHO Guidelines on Physical Activity and Sedentary Behaviour, 2024). If unable to meet the guidelines, people living with dementia should engage in as much PAE as their abilities permit.

Benefits of PAE can vary depending on the specific type of activity. Dance is both an art and a form of exercise that involves motor, cognitive, social, and emotional skills and it can improve balance, QOL and cognition (Charras et al., 2020). Dancing with others can strengthen social relationships for people living with dementia and their care partner, enhancing their quality of life (QOL) (Wu et al., 2015). Group dance can positively effect mood and wellbeing of PLWD, it provides social engagement and psychological relaxation (Fong Yan et al., 2024; Wang, Liu, et al., 2022). Céilí dance is a traditional form of social Irish dance, it is a partnered dance and it involves structured group movements performed to Irish music (Foley, 2011). An Irish population will likely be familiar with this style of dance as it is a popular form of dance in Ireland (Clifford et al., 2019). When participants are familiar with the activity, it can potentially help them to reconnect with their bodies thus allowing access to past experiences and memories (Hamill et al., 2012).

There are barriers to PAE for people living with dementia and care partners, including physical abilities and low motivation (Vseteckova et al., 2020). Understanding their needs is important, as it may help overcome the barriers, and increase participation (van Alphen et al., 2016). There is currently limited exercise interventions developed for people living with dementia and care partners (Park & Cohen, 2019). A tailored intervention may allow for increased participation and effectiveness (Pitkälä et al., 2013).

Intervention development should be guided by evidence supported tools to enhance the success of the intervention. The Medical Research Council Framework (MRCF) is the gold standard for intervention development (Shahsavari et al., 2020). It outlines four steps; development, feasibility, evaluation and implementation, enabling researchers to identify the most appropriate methodology for their intervention (Shahsavari et al., 2020). Additionally, interventions targeting behaviour change are often guided by the Behaviour Change Wheel Framework (BCW) (Michie et al., 2011). The BCW incorporates 19 behaviour change frameworks (Webb et al., 2016). Changing behaviour requires three essential factors: capability, opportunity and motivation to perform the behaviour (COM-B) (Michie et al., 2011). The BCW identifies the components needed to perform the selected behaviour (Barker et al., 2016). The BCW supports the development of effective and feasible interventions across populations (Cantwell et al., 2020; Kwok et al., 2021).

Patient and public involvement (PPI) is a crucial component in developing interventions, it enhances the research quality, relevance and value (Biggane et al., 2019; Brett et al., 2014; Patient and Public Involvement in Research (PPI) - HSE Research & Development, 2021). In dementia research, patient and public involvement aids the development of an evidence base and advises on the process of future research (Howard Wilsher et al., 2017). Previous dementia research has focused on care partners and healthcare professionals, excluding people living with dementia due to assumptions about their reliability (Gove et al., 2018). People with a lived experience (people living with dementia and those who care for them), provide essential insights to ensure authenticity and relevance (Roberts et al., 2020). Patient and public involvement allows an understanding from the perspective of the target population (van Schelven et al., 2020). In this study, PLWD and care partners were involved as collaborators in co-designing the intervention content and advising on recruitment and data collection processes.

This study aimed to demonstrate the development process of a dance exercise intervention for people living with dementia and their care partners within an established medically supervised community-based exercise program for people with chronic illnesses. The MRCF and the BCW guided the interventions development to ensure a patient centred approach. The target behaviour was to increase the physical activity levels of people living with dementia and care partners.

## Methodology

### Overview

This dance exercise intervention development followed the MRCF (Shahsavari et al., 2020) for developing complex interventions and used the BCW (Michie et al., 2011) to inform appropriate behaviour change techniques (BCT) (Truelove et al., 2020). The MRCF outlines four stages including, development, feasibility, implementation and evaluation of complex interventions. The development stage of the MRCF involves three guiding processes, as seen in Table 1. These are; (1) identify the existing evidence, (2) develop a theory and (3) model the process and its outcomes (Craig et al., 2008). The BCW offers a structured approach to intervention design by applying the COM-B model to identify behavioural influences, linking them to appropriate intervention functions, and ultimately mapping these functions to relevant BCT (Michie et al., 2011, 2013). The three guiding process of the development stage of the MRCF were further broken down to embed the steps of the BCW (Table 1). In addition, a participant advisory group (PAG) was assembled to ensure the perspectives of people living with dementia, and care partners, were involved throughout the research process. The reporting of this study followed the GUIDED checklist (see Supplemental File 1). This research ethics was approved by The Technological University of the Shannon Research Committee, the reference number was 2023/06/8. All respondents gave either written or verbal consent before participating in this research.

**Table 1.** Alignment of the Development Processes and the Behaviour Change Wheel

Development stages	Steps of study
1. Identify existing evidence	1.(a) Define problem and select target behaviour 1.(b) Specify the target behaviour 1.(c) Identify what needs to change
2. Develop theory	2.(a) Align gathered data with COM-B constructs 2.(b) Identify intervention functions. 2.(c) Identify behaviour change techniques
3. Model process and outcomes	3.(a) Develop draft intervention components 3.(b) Engage with stakeholder panel 3.(c) Finalise intervention components

### Participant Advisory Group

A PAG was established through the Dementia Research Advisory Team in the Alzheimer’s national governing organisation (NGO) at the beginning of the study. The PAG consisted of two people living with dementia and two care partners who volunteered to participate in this research advisory team. All meetings were conducted virtually using Zoom. The PAG were involved in two specific phases of the study; (1) Reviewing the interview and focus group topic guides and (2) The final review of the intervention components. Their feedback was gathered and implemented at both stages. The PAG collaborated on the co-design of the intervention content, recruitment and data collection processes.

### Procedure

#### Stage 1. Identify Existing Evidence

##### Define Problem and Select Target Behaviour

A general search of research articles, online books and grey literature within the area of PAE for people living with dementia and care partners, PAE for older adults and different forms of PA for older adults was conducted. Increasing PA in people living with dementia and care partners was identified as the primary target behaviour (Lindsay et al., 2022; van Alphen et al., 2016).

##### Specify the Target Behaviour

The target behaviour was further specified by using 6 questions as advised in the BCW steps (Michie et al., 2011). The questions included: (1) Who needs to perform the behaviour? (2) What does the person need to do differently to achieve the desired change? (3) When will they do it? (4) Where will they do it? (5) How often will they do it? (6) With whom will they do it? The responses were informed by the literature and qualitative findings.

##### Identify What Needs to Change

To address the target behaviour, a multi-method approach was utilized. This included a literature review, interviews and focus groups. Key statements from the literature review were generated to represent the findings of this phase.

A mix of both semi – structured focus groups and individual interviews were conducted with people living with dementia and care partners where their perspectives and experiences of PAE were discussed. The focus groups and interviews followed a semi-structured topic guide, with slight variations in guides used with people living with dementia and care partners (see Supplemental File 2). Each construct of the COM-B model (Michie et al., 2011) (capability, opportunity and motivation) was addressed when developing the interview and focus group guides, to identify the constructs in most need of support. The topics included in the topic guides were, Understanding of PA, Previous and Current levels of PA, Important Factors of PA for PA interventions. The PAG reviewed the topic guides and gave their feedback on the topic guides. Their feedback was implemented to finalise the topic guides.

Participants for the focus groups and interviews were recruited though several avenues. A recruitment email and flyer were circulated to relevant organisations and individuals (e.g. relevant NGO, Exercise groups, Memory Clinics). The flyer was shared on social media. In-person visits to relevant groups were made by the researcher (Dementia Cafes, community-based exercise classes, social clubs,). Engagement with healthcare professionals, where the researcher provided details of the study, were conducted as part of the recruitment strategy. All interested persons were asked to complete an expression of interest form on Microsoft Forms or to contact the researcher whose details were provided. Prior to taking part all interested individuals were provided with a participant information sheet and completed an informed consent form or informed assent with a proxy informed consent if required.

Semi – structured focus groups (n = 3) and interviews (n = 14) took place online and in person. Each focus group consisted of four care partners. The focus groups and interviews were between 20–90 minutes (average = 34 minutes, standard deviation ±16 minutes). All were audio recorded via zoom or using a mobile recording device and transcribed verbatim. The transcripts were analysed using content analysis, via the analysis software NVivo 12. Content analysis provided the researcher with a process to identify patterns and key points within the gathered information (Vears & Gillam, 2022).

#### Stage 2. Develop Theory

##### Align Gathered Data with COM-B Constructs

Key findings and recommendations from the literature and qualitative data were summarised and were aligned with the COM-B model to identify which COM-B constructs should be targeted. The COM-B constructs include: physical capability, psychological capability, physical opportunity, social opportunity, automatic motivation and reflective motivation.

##### Identify Intervention Functions

This phase involved identifying which intervention functions were relevant to support the selected COM-B model constructs. The BCW framework describes nine intervention functions, training, modelling, incentivisation, environmental restructuring, education, persuasion, coercion, enablement and restrictions, to guide intervention development (Michie et al., 2011).

##### Identify Behaviour Change Techniques

There are ninety - three behaviour change techniques (BCTs) in the behaviour change technique taxonomy. All the BCTs were reviewed. The most applicable BCTs were selected based on their alignment with the intervention functions. The affordability, practicability, effectiveness, acceptability, safety and equity (APEASE) criteria was used to evaluate each BCT (Michie et al., 2011).

#### Stage 3. Modelling Process and Outcomes

##### Develop Draft Intervention Components

The initial draft of the intervention components was created by translating the selected BCTs into practical, deliverable content.

##### Engage with Stakeholder Panel

The final stage involved engagement with an expert stakeholder panel, including healthcare professionals and exercise professionals who worked with people living with dementia or who worked in the exercise and dance industry (n = 8). All stakeholders were recruited via email. The stakeholder panel discussion was held online via Zoom and lasted 1 hour and 16 minutes. The developed intervention components were presented and facilitated a group discussion based on their experiences. The discussion was transcribed. Stakeholder feedback was summarized into additional comments and recommendations incorporated into the intervention components.

##### Finalise Intervention Components

An updated version of the intervention components was presented to the PAG. The PAG discussed their thoughts on the practicality, accessibility and relevance of the developed intervention. Conclusive changes were made to the components based on their input, and the intervention was finalised.

## Results

### Stage 1. Identify Existing Evidence

#### Define Problem and Select Target Behaviour

The researchers identified the problem as follows: people living with dementia and their care partners face a high risk of low PA levels (Lindsay et al., 2022; van Alphen et al., 2016). Despite understanding the physical and psychological benefits of PAE, people living with dementia and care partners have low PA levels (Lambert et al., 2016; van Alphen et al., 2016). Evidence supports dance in particular as a form of PAE as it can benefit people living with dementia’s physical functioning, their mood and their social engagement, all of which positively influence their quality of life (Mabire et al., 2019; Wang, Song, & Niu, 2022; Wu et al., 2021). As a result, the target behaviour was to increase physical activity levels via a dance exercise intervention for people living with dementia and their care partners. Further evidence is provided in Supplemental File 3.

#### Specify the Target Behaviour

The intervention’s target behaviour, to increase levels of PAE for people living with dementia and care partners through a dance exercise intervention, is specified in detail in Table 2.

**Table 2.** Alignment of Target Behaviour Specifications With Behaviour Change Wheel Criteria

Target behaviour specifications	
What is the target behaviour?	To increase the physical activity levels of people living with dementia, and care partners of people living with dementia, through a dance exercise intervention.
Who needs to perform this behaviour?	People who have been diagnosed with dementia and care partners of people living with dementia.
What do they need to do differently?	Begin attending a community-based dance exercise class.
When will they do it?	Morning to early afternoon.
Where will they do it?	In a community based, accessible hall.
How often?	Participants will take part in a class twice a week.
With whom?	Participants will take part with fellow class participants and may attend with their care partner/family member/friend.

#### Identify What Needs to Change

A multi-method approach to address the target behaviour yielded results in the form of literature review statements and focus group/interview recommendations. The literature review identified nine key studies that further informed the intervention development. These studies focused on the physical and psychological effects of PA, exercise and dance for older adults, people living with dementia and care partners and previous interventions in this population. Table 3 provides an overview of the 13 keys literature review statements that guided the intervention development procedure.

**Table 3.** Literature Review Statements

	Statements generated from literature review	Source (First author, year)
1	Despite PA health benefits, people living with dementia’s PA levels are low, lower than those of the same age who do not have dementia.	van Alphen et al. (2016)
2	Caring can have a negative impact on the physical and psychological health of the care partner.	Lambert et al. (2016)
3	Physical inactivity can lead to further decline in physical functioning.	Lam et al. (2018)
4	PAE can improve strength, balance, walking speed and endurance, and mobility in people living with dementia.	Liu et al. (2021)
5	Dance is a multi-modal activity involving motor skills, cognitive and sensorimotor capacities, emotional and social aptitudes and sensory abilities	Mabire et al. (2019)
6	Importantly for people living with dementia, dance is a holistic intervention offering benefits including socio-psychological wellbeing, social engagement and enjoyment.	Wu et al. (2021)
7	Dance based interventions can improve neuropsychiatric symptoms, such decreasing psychological distress in people with depression and anxiety, and improving their QOL, interpersonal and cognitive skills.	Wang, Song, & Niu, (2022), Wang, Liu, et al. (2022)
8	Care partners play a key role in promoting PAE for people living with dementia.	van Alphen et al. (2016)
9	PAE can help care partners maintain their health to allow them to successfully perform their vital roles.	Lambert et al. (2016)
10	Multimodal exercise with resistance, aerobic, balance, flexibility and functional training for 60 minutes 2–3 times a week is effective in improving various aspects of physical function such as mobility balance and strength.	Lam et al. (2018)
11	Goal setting and action planning can be a motivating factor in maintaining PAE for people living with dementia through self-motivation.	Nyman et al. (2018)
12	Group exercise gives opportunity of social interaction and inclusion	Mabire et al. (2019)
13	Participation in shared activities between the people living with dementia and their care partner may promote positive outcomes amongst them.	Petts & Urmston (2022)

Twenty-six individuals participated in the focus groups and interviews (69.2% female, 30.7% male). This included seventeen care partners (82.4% female, 17.6% male) and nine people living with dementia (44.4% female, 55.5% male). Each focus group had four care partners. Additionally, five care partners and nine people living with dementia participated in individual interviews. Eight key recommendations were established from the qualitative data (Table 4).

**Table 4.** Interview and Focus Group Recommendations

	Focus group/Interview recommendation
1	Mixed population group - to include people that do not have dementia. This may help to eliminate the stigma that is often associated with dementia
2	Logistics - to have suitable logistics for the class for people living with dementia, such as having the class in the late morning, early afternoon, ensuring it is in an accessible location, and the class takes place no more than twice a week
3	Exercise variety - including a variety of exercises during the class was identified as an important element, so that not just dance was included, but activities such as balance and strengthening exercises.
4	Social support – encouraging people living with dementia to attend with a care partner or family member. Allowing participants to socialize before or after class and get to know others in similar circumstances.
5	Inclusive exercise/dance – to allow a range of abilities to participate in the class was critical.
6	Clear instruction – providing clear instructions on how to perform the different exercises and movements, (to help participants feel more comfortable) may increase and maintain participation and attendance.
7	Class reminders – a text or call to each participant the morning of the intervention sessions to remind and encourage the participants to attend was highlighted as an important tool to support participants.
8	Keep it fun and enjoyable - ensuring a fun and enjoyable atmosphere for the class would encourage continuous attendance and participation.

### Stage 2. Develop Theory and Intervention Functions

The development of the intervention components followed a process of identifying COM-B constructs (2 (a)), which were mapped to BCW functions (2 (b)) and finally suitable BCTs to embed into the intervention were identified (2 (c)).

#### Aligning Gathered Data with COM-B Constructs

The COM-B constructs requiring action in this population, as identified through focus groups, interviews, and the literature review, were physical and psychological capability, along with physical and social opportunity (Table 5). In addition, the literature review also highlighted autonomic and reflective motivation as constructs to be included in intervention development for this population (Table 5). (Systematic Review of Behaviour Change Techniques to Promote Participation in Physical Activity among People with Dementia - Nyman - 2018 - British Journal of Health Psychology - Wiley Online Library, 2024)

**Table 5.** Mapping of BCW Constructs to Literature Review Statements and Interview and Focus Group Recommendations

COM-B constructs	Literature review statements	Interview and focus group recommendations
Physical capability	2 (Lambert et al., 2016), 3 (Lam et al., 2018), 4 (Liu et al., 2021), 5 (Mabire et al., 2019), 6 (Wu et al., 2021), 9 (Lambert et al., 2016), 10 (Lam et al., 2018)	3, 5,
Psychological capability	2 (Lambert et al., 2016), 5 (Mabire et al., 2019), 7 (Wang, Song, & Niu, 2022), 9 (Lambert et al., 2016)	5, 6, 7
Physical opportunity	8 (van Alphen et al., 2016), 9 (Lambert et al., 2016), 10 (Lam et al., 2018),	2, 4, 7
Social opportunity	5 (Mabire et al., 2019), 6 (Wu et al., 2021), 7 (Wang, Song, & Niu, 2022), 12 (Mabire et al., 2019), 13 (Petts & Urmston, 2022)	1, 4, 7
Automatic motivation	1 (van Alphen et al., 2016)	
Reflective motivation	4 (Liu et al., 2021), 8 (van Alphen et al., 2016), 11 (Nyman et al., 2018), 13 (Petts & Urmston, 2022)	

#### Identify Intervention Functions

Six of nine possible intervention functions were selected based on the BCW framework guidelines, to support the selected COM-B constructs (Michie et al., 2011). These included education, enablement, training, incentivisation, modelling and environmental restructuring. The three intervention functions excluded—persuasion, coercion, and restriction—were deemed inappropriate for this intervention.

#### Identify Behaviour Change Techniques

Using the APEASE criteria, twenty–one of a possible ninety–three established BCTs within the BCW framework were selected. These twenty – one BCTs were considered the most appropriate and compatible with the identified intervention functions (education, enablement, training, incentivisation, modelling and environmental restructuring). This can be seen in Table 6.

**Table 6.** Behaviour Change Techniques Alignment With APEASE Criteria

Intervention functions	Behaviour change techniques	Application	Do BCT meet the APEASE criteria in context of the behaviour?
Education	5.1. Information about health consequences	Talk from doctor during induction.	Yes, it is acceptable, practical, affordable, safe and effective. Provides participants with additional benefits and possible risks of exercise.
	5.6. Information about emotional consequences	Talk from doctor during induction.	Yes, it is acceptable, practical, affordable, safe and effective.
	2.1. Monitoring of behaviour by others without feedback	Record attendance, and how they are getting on during class.	Yes, it is acceptable, practical, affordable and safe.
	9.1. Credible source	Support from exercise staff and GPs. Referrals from healthcare professionals.	Yes, it is acceptable, practical, affordable, safe and effective. The referral from a trusted professional increases acceptability and safety.
Enablement	Goal setting (behaviour)	1 month goal setting to encourage physical activity and exercise.	Yes, it is acceptable, affordable, safe and effective. It may be easier to continue PAE when a goal has been set.
	1.3. Goal setting (outcome)	1 month goal setting to encourage physical activity and exercise.	Yes, it is acceptable, affordable, safe and effective. It may encourage PAE if they focus on an outcome they will see and benefit from.
	1.4. Action planning	1 month action planning to encourage physical activity and exercise.	Yes, it is acceptable, affordable, safe and effective. Planning how to achieve their goal will encourage meeting it.
	1.5. Renew behaviour goals	3- And 6-month goal setting.	Yes, it is acceptable, affordable, safe and effective. Allows practical adjustments to be made for each individual.
	1.7. Review outcome goals	3- And 6-month goal setting.	Yes, it is acceptable, practical, affordable, safe and effective. Allows practical adjustments to be made for each individual.
	3.1. Social support (unspecified)	Encouragement to attend class, prompts used as reminder.	Yes, it is acceptable, practical, affordable, safe and effective. Encourages participants to continue attendance.
	3.2. Social support (practical)	Invite care partner/family member to attend with people living with dementia, help them to attend (encouragement/transport).	Yes, it is acceptable, affordable, safe and effective. The practicality may depend on each individual but it is not mandatory.
	3.3. Socias Support (emotional)	Invite care partner/family member to attend with people living with dementia.	Yes, it is acceptable, affordable, safe and effective. The practicality may depend on each individual and their relationship with care partner/family member.
Training	2.1. Monitoring of behaviour by others without feedback	Record attendance, and how they are getting on during class.	Yes, it is acceptable, practical, affordable and safe.
	2.5. Monitoring of outcomes of behaviour without feedback	Observe and record testing at baseline, 3 and 6 months.	Yes, it is acceptable, practical, affordable, safe and effective. May provide motivation to see improvement and maintenance.
	4.1. Instruction on how to perform behaviour	Exercise instruction given by instructor to class.	Yes, it is acceptable, practical, affordable, safe and effective. May further understanding of behaviour and acceptability of the behaviour.
	15.1. Verbal persuasion about capability	Encouragement from instructor.	Yes, it is acceptable, practical, affordable, safe and effective.
Incentivisation	2.7. Feedback on outcomes of behaviour	Provide feedback to participants on testing and movement.	Yes, it is acceptable, practical, affordable, safe and effective. May provide motivation to continue to see improvements and maintenance.
Modelling	6.1. Demonstration of behaviour	Instructor demonstrates before each exercise.	Yes, it is acceptable, practical, affordable, safe and effective. Allows participants
Environmental restructuring	7.1. Prompts/Cues	Text/call to participants on day of class/testing.	Yes, it is acceptable, practical, affordable, safe and effective.
	8.3. Habit formation	Continuing twice a week for 6 months.	Yes, it is acceptable, affordable, safe and effective. The practicality may vary for each individual but allows attendance at least one class and allows rest between classes.
	12.5. Adding objects to environment	Céilí dance class in local area.	Yes, it is acceptable, practical, affordable, safe and effective.

### Stage 3. Modelling Process and Outcomes

#### Developed Draft Intervention Components

The twenty-one selected BCTs were developed into a preliminary intervention framework which included six components (Table 7). These components included; Supervised Céilí dance exercise class, Induction session, Assessments of physical and psychological health, Goal setting and action planning session, Daily prompts for class, and Social aspect. Literature review statements and qualitative recommendations were used to translate the intervention framework into six practical and deliverable intervention components. The timing of the assessments (Liu et al., 2021; Nyman et al., 2018; Wang, Song, & Niu, 2022), the goal setting and action planning (Mabire et al., 2019; Wu et al., 2021) were recommended in previous literature. The daily prompts and social aspect were important points that were emphasised in the gathered qualitative data. The supervised Céilí dance exercise class, the induction, and the assessment of both physical and psychological health followed the standard practice of the established community based medically supervised exercise service. Table 8 outlines the BCW intervention functions and the COM-B constructs that align with each intervention component.

**Table 7.** Mapping of the Intervention Components to the BCTS

Behaviour change techniques	Supervised dance exercise class	Induction session	Assessments of physical and psychological health	Goal setting and action planning session	Daily prompts for class	Social aspect
1.1 goal setting (behaviour)	X			X		
1.3 goal setting (outcome)				X		
1.4 action planning				X	X	
1.5 review behaviour goals				X		
1.7 review goal (outcome)			X	X		
2.1 Monitoring of behaviour by others without feedback	X	X				
2.2 Feedback on behaviour	X	X	X			
2.5 Monitoring of outcomes of behaviour without feedback	X					
2.7 Feedback on outcomes of behaviour	X		X			X
3.1 Social support (unspecified)	X	X			X	X
3.2 Social support (practical)	X	X			X	X
3.3 Social support (emotional)	X	X			X	X
4.1 Instruction on how to perform the behaviour	X	X	X			
5.1 Information about health consequences		X				
5.6 Information about emotional consequences		X				
6.1 Demonstration of the behaviour	X	X	X			
7.1 Prompts/Cues	X				X	X
8.3 Habit formation	X				X	X
9.1 Credible source		X				
12.5 Adding objects to the environment	X					
15.1 Verbal persuasion about capability	X	X	X	X

**Table 8.** Mapping of the Intervention Components to the COM-B Constructs and the BCW Intervention Functions

Intervention components	BCW intervention functions	COM-B constructs
Supervised céilí dance exercise class	Education, enablement, training, incentivisation, environmental restructuring, modelling	Capability – Physical and psychological Opportunity – Social and physical Motivation - automatic
Induction session	Education, enablement, incentivisation, modelling	Capability – Physical and psychological Opportunity – Physical and social
Assessments of physical and psychological health	Education, enablement, training, incentivisation, modelling	Capability – Physical and psychological Opportunity – Physical and social
Goal setting and action planning session	Education, enablement, training	Capability – Physical and psychological Motivation - reflective
Daily prompts for class	Education, incentivisation, training, environmental restructuring	Opportunity – Physical and social Motivation – Automatic
Social aspect	Incentivisation, environmental restructuring	Opportunity – Social Motivation – automatic

#### Engagement with Stakeholder Expert Panel

The panel consisted of healthcare professionals (n = 4) and exercise professionals (n = 4) who work with people living with dementia or who are in the exercise and dance industry (Geriatric consultants, dementia nurse specialists, physiotherapists, exercise instructors, Irish dance teachers and academics). The stakeholder panel agreed that the presented intervention was acceptable and would benefit the participants physical, psychological, and social health. Several recommendations were made to optimise the developed intervention (Table 9). These recommendations included emphasising current components of the intervention including a social aspect after the class, ensuring there is breaks throughout the class and keeping the movements and exercises adaptable. Other recommendations suggested a heightened awareness of the terminology used when giving instructions, to ensure participant safety. For example, when describing a step in a dance, using turn around the other couple with your partner rather than spin around the other couple with your partner. The action may be interpreted differently depending on the terminology. The panel voiced concerns about attendance and adherence, they suggested possible solutions, including the time of the class. They echoed the preferences of the people living with dementia and care partners, suggesting a start between 11am –2pm. Another approach to support attendance was to issue a reminder via call or text to the participants on the morning of the class.

**Table 9.** Stakeholder Expert Panel Recommendations

Stakeholder recommendations	
**1. Awareness of terminology** Be aware of the instructions are phrased. Instructions can be interpreted differently e.g. a spin sounds like it needs to be a fast movement, therefore changing it to a turn around.	‘Sometimes physical ability doesn’t match confidence’ – P4 (dance and exercise academic specialist) ‘we’re going to maybe invent other ways that we can move or rotate really slowly because otherwise the if there’s high energy music it can lead.’ - P4 (dance and exercise academic specialist)
**2. Attendance and adherence** Suggestions included a suitable time to attend, 11am-2pm was agreed as a good timeframe. Sending a reminder on day of class e.g. text or call participants. To ensure participants are aware of the class, time and location**.**	‘I think routine as well is hugely important… morning classes will probably work better for you then as well’ – P1 (physical activity and exercise academic specialist)
**3. Social aspect** An extra social aspect of the intervention, including time for a cup of tea after the class was met by enthusiasm from the stakeholder panel. It may be beneficial to care partners and people living with dementia. Gives the opportunity to speak with others in similar situations to themselves, to socialise outside of their home and gives everyone something to look forward to at the end of the class.	‘A lot of people are recently coming from the classes…saying to me that the biggest thing they’ve noticed is a social aspect…everyone seems to love it’ – P3 (health and exercise specialist)
**4. Water breaks** Ensure and encourage water breaks throughout class so participants are hydrated throughout the exercise.	‘I would build in breaks and encourage them… it’s quite high intensity’ - P4 (dance and exercise academic specialist)
**5. Adaptable exercises/movements** Awareness of participant’s abilities, ensure the exercises can be adapted for all participants e.g. supported exercises. Many people living with dementia have comorbidities which may impact their abilities.	‘In terms of like having a seated or supported component because there might be other, conditions apart from dementia’ – P4 (dance and exercise academic specialist)

#### Finalise Intervention Components

The proposed intervention was presented to the PAG. Final recommendations were provided on each intervention component and the intervention in its entirety.

Monitoring of attendance, contact with participants on the day of the intervention, encouraging peer support and encouragement from instructors was suggested to support attendance and adherence to the intervention. The location of the intervention was mentioned as a possible concern as it was not accessible by public transport. Unfortunately, this could not be changed. Providing financial support for transport e.g., a taxi, was the suggested mediation. The PAG accepted the intervention design and its components. An overview of the final intervention components is provided in Table 10.

**Table 10.** The Components of the Céilí Dance Exercise Intervention

Intervention components	Description
Supervised céilí dance exercise class	A 60-min instructor led céilí dance exercise class to be attended twice weekly for 6 months was developed. The class components include a warm-up, céilí dance component, a strength component, balance component and a cool down. The class was facilitated by a qualified Irish dance teacher and exercise instructors.
Induction session	Participants will be invited to an induction session after being referred by a healthcare professional. The induction involves a talk about the benefits of exercise and taking part in an exercise programme from a medical doctor. Physical and psychological health measures will be gathered from participants including; 6-min walk test, dual task timed up and go, adapted sit and reach test, 5 repetition sit to stand, dual task gait assessment, handgrip test. Questionnaires including quality of life, positive and negative mood, fear of falling, a pain scale. The PA levels of the participants will be measured using an ActivePAL monitor.
Assessments of physical and psychological health	Physical and psychological health assessments are to be repeated at 3 months and 6 months post baseline. Each participant will be invited to a post intervention focus group to allow them to share their experience and opinions of the intervention.
Goal setting and action planning session	A goal setting and action planning session will be conducted with each participant 1 month post baseline and this will be revisited at 3- and 6-months post baseline.
Daily prompts for class	A reminder text or phone call will be made to each participant on the morning of the classes and re-testing including the time and location of the intervention.
Social aspect	The social aspect will be involved in various ways. The people living with dementia are encouraged to attend with their care partner or a family member. There will be tea and coffee held after one of the classes each week. The exercise class is a group session, and all the dances are partnered dance.

## Discussion

This study outlines the methodology used to develop a patient-centred dance exercise intervention for people living with dementia and their care partners. The aim of the intervention was to increase or maintain levels of PAE for this population. Interventions focused on PAE have significant potential to benefit people living with dementia and care partners through their physical and mental health including their ADL and QOL (Lambert et al., 2016; Liu et al., 2021; Petts & Urmston, 2022). The study employed the MRCF and the BCW to guide the development of an appropriate dance exercise intervention for the selected population. Based on the evidence from the literature review and the qualitative investigation, an intervention composed of six components was developed and linked to the COM-B constructs and BCW intervention functions. The intervention components include; a supervised dance exercise class, an induction session, assessments of physical and psychological health, a goal setting and action planning session, daily prompts for class and the social aspect.

Theoretically informed interventions may increase the efficacy of exercise-based behaviour change interventions (Charras et al., 2020). This intervention was developed by applying steps from the MRCF and the BCW framework (Michie et al., 2011; Shahsavari et al., 2020). The resulting intervention programme was evidence-based and specific to the needs of people living with dementia and care partners. The evidence base was gathered via a literature review. Focus groups and interviews with people living with dementia and care partners were conducted to understand the PAE perspectives and experiences of people living with dementia and care partners. Communicating with the target population was the best way to consciously involve their perspective and experiences with the hope of increasing attendance and adherence to this intervention (Bielinska et al., 2022). The BCW has previously supported the development of multiple PAE for various populations with specific requirements (Cantwell et al., 2020; Chen et al., 2022).

Studies for people living with dementia have not consistently engaged with people living with dementia in the development stages of research (Brooks et al., 2017). This study involved interviews and focus groups with people living with dementia and care partners, to understand their perspectives and what they want and require from a PAE intervention. Previously, care partners and healthcare professionals have been asked to give their opinions on behalf of this population. They have been asked to give proxy answers rather than involving people living with dementia (Ho et al., 2018). This emphasises the importance of involving people living with dementia, care partners and healthcare professionals at various points of the development to ensure different viewpoints are included. Combining methods qualitative data collection and PPI allows the intervention to be developed with insight into behaviour and this increases the quality and acceptability of the intervention (Biggane et al., 2019).

This study enlisted the expertise of a PAG, comprised of people living with dementia and care partners. The PAG gave their opinions on the research being conducted and implemented for the intervention development. Involving people living with dementia and care partners as part of the development process through PPI, assured the lived experience was included throughout the project. Establishing a PAG is becoming standard practice when engaging with diverse populations (Koskinas et al., 2022). The PAG in this study worked with the researchers to finalise the topic guides for the focus groups and interviews. This established all questions were appropriate in terms of content and language when engaging with people living with dementia and care partners. They discussed the developed intervention components with the researchers. Each component was considered in terms of practicality and reasoning. Possible barriers to attending the intervention were debated and potential feasible solutions were determined. This approach is increasingly valued because involving PPI has been shown to enhance both the research process and its outcomes. It further enhances the creditability and relevance of the research (Gove et al., 2018).

The qualitative data from this study highlighted the request from the people living with dementia and the care partners for a community-based, mixed population class. The social element of PAE is of particular importance for people living with dementia, as staying social and active within their community is imperative for good social and mental health (Pakstis et al., 2018). The social aspect of exercising in a group has been reported to add enjoyment to PAE for the participants (Fox et al., 2007). This shows there is a recognized need for interventions that incorporate social engagement to support individuals with dementia. Good social health positively influences both physical and psychological well-being (Joyce et al., 2022). Older adults with high participation in social activities in community groups, including PAE and non PAE based, experienced better QOL, higher self-esteem and stronger emotional and psychological support, along with greater muscle mass, better balance and improved cognition than those with low social participation (Sepúlveda-Loyola et al., 2020).

Previous evidence has stated despite the benefits, people living with dementia and care partners have low levels of PAE engagement (van Alphen et al., 2016). This is due to insufficient suitable support systems for this population in both rural and urban areas (Hobson et al., 2019). The results from the qualitative data in this study show people living with dementia, care partners and healthcare professionals understand the of importance of PAE for their health. One of the primary barriers to their PAE is the lack of tailored services. This emphasises the importance of developing a person-centred PAE intervention for people living with dementia and care partners.

Exercise and dance interventions have previously demonstrated increases in positive mood, improvements in agitation, anxiety and overall wellbeing (Bennett et al., 2021; Koh et al., 2020). The developed intervention from this study has the potential to positively effect people living with dementia and their care partners due to its patient-centred approach. The intervention is community based, it includes a social aspect and focuses on dance which is a social form of exercise. These points may motivate and support other psychological factors of the participants. This social element is vital for people living with dementia within their community as it reduces isolation and fosters social interaction with others (Cooper & Thomas, 2002). There is a need for PAE intervention for people living with dementia and care partners as they can have a positive impact on their life.

### Strengths and Limitations

This study utilized the effective and feasible methodologies of intervention design by implementing the use of the MRCF and the BCW framework (Michie et al., 2011; Shahsavari et al., 2020). These frameworks use a structured evidence-based approach while allowing adaptation and flexibility for different populations and styles of interventions. The sample of participants involved in the focus groups and interviews were previously involved in PAE throughout their lives and therefore may have had focused on a more positive outlook on PAE than the wider population. This may be because this sample of people were more willing to speak about their previous PAE experiences due to their positive nature. The recruitment timeline was long for the number of participants recruited. The recruitment of people living with dementia for the interviews and focus groups resulted in lower numbers than expected for the qualitative analysis.

## Conclusion

This study aimed to develop a relevant, effective, person-centred dance exercise intervention for people living with dementia and care partners by integrating developmental theory and practice. The intervention included six intervention components: a supervised dance exercise class, an induction session, assessments of physical and psychological health, a goal setting and action planning session, daily prompts for class, and a social aspect. These components structure a dance exercise intervention intending to increase the physical activity levels of people living with dementia and care partners. The next step involves implementing and evaluating this intervention in a practical setting. Future research should assess the feasibility and effectiveness of implementing this type of intervention with people living with dementia and care partners.

## Supplemental Material

Supplemental Material - The Development of the Dance Smart Intervention: A Céilí Dance Exercise Intervention for People Living With Dementia and Their Care Partners (Dance Smart Intervention) Supplemental Material for The Development of the Dance Smart Intervention: A Céilí Dance Exercise Intervention for People Living With Dementia and Their Care Partners (Dance Smart Intervention) by Niamh Kelly, Fiona Skelly, Kieran Dowd, Desmond O’Neill, Noel McCaffery, Clare McDermott in Dementia.

## Data Availability

The dataset generated during the current study is not publicly available due to the involvement of a vulnerable population. Access of the data is restricted to safeguard the wellbeing of the participants.*
