# Supplementary material for: The Development of the Dance Smart Intervention: A Céilí Dance Exercise Intervention for People Living With Dementia and Their Care Partners (Dance Smart Intervention)
Source: Dementia (London). 2026 Mar 19;25(8):1939–61. doi: 10.1177/14713012261435732 (PMC13683915; doi:10.1177/14713012261435732)
Supplement: Supplementary file 1 [file sj-pdf-1-dem-10.1177_14713012261435732.pdf]

Supplementary File 1

| Item Description | Explanation | Page in manuscript where item is located |
|---|---|---|
| 1.Report the context for which the intervention was developed. | Understanding the context in which an intervention was developed informs readers about the suitability and transferability of the intervention to the context in which they are considering evaluating, adapting or using the intervention. Context here can include place, organisational and wider socio-political factors that may influence the development and/or delivery of the intervention (15). | 5 - 6 |
| 2. Report the purpose of the intervention development process | Clearly describing the purpose of the intervention specifies what it sets out to achieve. The purpose may be informed by research priorities, for example those identified in systematic reviews, evidence gaps set out in practice guidance such as The National Institute for Health and Care Excellence or specific prioritisation exercises such as those undertaken with patients and practitioners through the James Lind Alliance. | 6 |
| 3. Report the target population for the intervention development process | The target population is the population that will potentially benefit from the intervention – this may include patients, clinicians, and/or members of the public. If the target population is clearly described then readers will be able to understand the relevance of the intervention to their own research or practice. Health inequalities, gender and ethnicity are features of the target population that may be relevant to intervention development processes. | 5-6 |
| 4. Report how any published intervention development approach contributed to the development process | Many formal intervention development approaches exist and are used to guide the intervention development process (e.g. 6Squid (16) or The Person Based Approach to Intervention Development (17)). Where a formal intervention development approach is used, it is helpful to describe the process that was followed, including any deviations. More general approaches to intervention development also exist and have been categorised as follows (3):- Target Population-centred intervention development; evidence and theory-based intervention development; partnership intervention development; implementation-based intervention development; efficacy based intervention development; step or phased-based intervention development; and intervention-specific intervention development (3). These approaches do not always have specific guidance that describe their use. Nevertheless, it is helpful to give a rich description of how any published approach was operationalised. | 6 – 7 |
| 5. Report how evidence from different sources informed the intervention | Intervention development is often based on published evidence and/or primary data that has been collected to inform the intervention development process. It is | 6 - 9 |

| | | |
|---|---|---|
| development process. | useful to describe and reference all forms of evidence and data that have informed the development of the intervention because evidence bases can change rapidly, and to explain the manner in which the evidence and/or data was used. Understanding what evidence was and was not available at the time of intervention development can help readers to assess transferability to their current situation. | |
| 6. Report how/if published theory informed the intervention development process. | Reporting whether and how theory informed the intervention development process aids the reader's understanding of the theoretical rationale that underpins the intervention. Though not mentioned in the e-Delphi or consensus meeting, it became increasingly apparent through the development of our guidance that this theory item could relate to either existing published theory or programme theory. | 10 -13 |
| 7. Report any use of components from an existing intervention in the current intervention development process. | Some interventions are developed with components that have been adopted from existing interventions. Clearly identifying components that have been adopted or adapted and acknowledging their original source helps the reader to understand and distinguish between the novel and adopted components of the new intervention. | 6 |
| 8. Report any guiding principles, people or factors that were prioritised when making decisions during the intervention development process. | Reporting any guiding principles that governed the development of the application helps the reader to understand the authors' reasoning behind the decisions that were made. These could include the examples of particular populations who views are being considered when designing the intervention, the modality that is viewed as being most appropriate, design features considered important for the target population, or the potential for the intervention to be scaled up. | 6 - 7 |
| 9. Report how stakeholders contributed to the intervention development process. | Potential stakeholders can include patient and community representatives, local and national policy makers, health care providers and those paying for or commissioning health care. Each of these groups may influence the intervention development process in different ways. Specifying how differing groups of stakeholders contributed to the intervention development process helps the reader to understand how stakeholders were involved and the degree of influence they had on the overall process. Further detail on how to integrate stakeholder contributions within intervention reporting are available (19) | 9, 20 |
| 10. Report how the intervention changed in content and format from the start of the intervention development process. | Intervention development is frequently an iterative process. The conclusion of the initial phase of intervention development does not necessarily mean that all uncertainties have been addressed. It is helpful to list remaining uncertainties such as the intervention intensity, mode of delivery, materials, procedures, or type of location that the intervention is most suitable for. This can guide other researchers to potential future areas of research and practitioners about uncertainties relevant to their healthcare context. | 20 – 22 |
| 11. Report any changes to interventions required or likely to be required for | Specifying any changes that the intervention development team perceive are required for the intervention to be delivered or tailored to specific sub | 16 – 22 |

| | | |
|---|---|---|
| subgroups. | groups enables readers to understand the applicability of the intervention to their target population or context. These changes could include changes to personnel delivering the intervention, to the content of the intervention, or to the mode of delivery of the intervention. | |
| 12. Report important uncertainties at the end of the intervention development process. | Intervention development is frequently an iterative process. The conclusion of the initial phase of intervention development does not necessarily mean that all uncertainties have been addressed. It is helpful to list remaining uncertainties such as the intervention intensity, mode of delivery, materials, procedures, or type of location that the intervention is most suitable for. This can guide other researchers to potential future areas of research and practitioners about uncertainties relevant to their healthcare context | This will be reported in the implementation and evaluation of this intervention. |
| 13. Follow TIDieR guidance when describing the developed intervention. | Interventions have been poorly reported for a number of years. In response to this, internationally recognized guidance has been published to support the high quality reporting of health care? interventions5and public health interventions14. This guidance should therefore be followed when describing a developed intervention. | The TIDieR checklist will be used in the reporting of the implementation and evaluation of the intervention. |
| 14. Report the intervention development process in an open access format. | Unless reports of intervention development are available people considering using an intervention cannot understand the process that was undertaken and make a judgement about its appropriateness to their context. It also limits cumulative learning about intervention development methodology and observed consequences at later evaluation, translation and implementation stages. Reporting intervention development in an open access (Gold or Green) publishing format increases the accessibility and visibility of intervention development research and makes it more likely to be read and used. Potential platforms for open access publication of intervention development include open access journal publications, freely accessible funder reports or a study web-page that details the intervention development process. | |

GUIDED – a guideline for reporting for intervention development studies.

## Supplementary File 2

(i). Focus Group/Interview Topic Guide for people living with dementia

| | | |
|---|---|---|
| **(i)** | **Understanding of Physical Activity** | |
| | Why don’t you start by telling me about your feelings about physical activity? | Positive, negative, why |
| **(ii)** | **Previous Levels of Physical Activity** | |
| | What was your physical activity like before being diagnosed with dementia? | |
| | What things led you to participate in physical activity? | |

| | | |
|---|---|---|
| **(iii)** | **Current Levels of Physical Activity** | |
| | Has anyone spoken to you about your physical activity as part of your care?<br>Who was it?<br>What did they recommend? | Doctor, physio, OT |
| | What opportunities have you had to participate in any exercise classes or groups since being diagnosed with dementia? | Someone to go with |
| | What are your physical activity levels like now?<br>Types of activities<br>How many times a week<br>Intensity (out of breath, can hold a conversation) | |
| **(iv)** | **Important Factors of Physical Activity** | |
| | What things would influence your decision to take part in an exercise class?<br>Can you tell me more about that?<br>How do you feel about that?<br>Motivators/barriers | Interests?<br>Friends?<br>Health?<br>Fun?<br>Time/cost/transport |
| **(v)** | **Preferences for Physical Activity Interventions** | |
| | Would you be interested in taking part in an exercise class?<br>kind of physical activity would you be interested in?<br>Type of physical activity you would enjoy?<br>How often would you like to take part per week?<br>Length of session? | Why? Why not?<br>Dance?<br>1/2/3 per week<br>1 hour |
| | We are thinking of developing a dance intervention<br><br>** PHOTOS OF PEOPLE DANCING**<br>I have brought a few photos with me today, of people dancing. I am hoping to hear what you think of these photos.<br>When you look at the photos how does it make you feel?<br>Does the photo remind you of anything?<br>What is your favourite photo and why? | Why? |
| | Would you have an interest in dance?<br>What kind of dance would you enjoy?<br>Why would you not be interested in dance? | |
| | How would you like to exercise?<br>Group/individual (Size class)<br>With care partner/partner/friend<br>With others who have dementia (only/other chronic illnesses) | |
| | How would you feel about an exercise class with your care partner?<br>Full class together<br>Mixed class, some exercise with care partner some without<br>Separate classes | |
| | If you had an opportunity to take part in a dance class with your care partner, would you go? | |
| | Would you prefer to be in a class with others who have older adults with chronic illnesses (dementia) only or a general | |

| | | |
|---|---|---|
| | public exercise class? | |
| | Class setting considerations:<br>What kind of setting should a class take place?<br>What time of the day would be best?<br>How would you get to the class?<br>What is an acceptable cost for a class? | Community hall<br>Morning/afternoon<br>By car/bus |
| | Class structure considerations<br>What kind of exercise class would you like to participate in?<br>(one type of exercise, mixed exercise classes) | |
| | What things would be important to you for taking part in exercise? | Must be fun?<br>Type of exercise |
| | Is there anything you would like to ask me? | |

(ii) Focus Group/Interview Topic Guide for care partners of people living with dementia

| | | |
|---|---|---|
| | | |
| | I'm going to start by asking you a little about the physical activity levels of the person you have cared for.<br>How would you describe their physical activity levels?<br>Has anyone spoken to you about p.a. as part of their care?<br>Who was it?<br>What was recommended? | Low, high, everyday things, shopping, walking around the house, walks, gardening, or exercise |
| | In relation to the person that you care for:<br>What do you think influences their physical activity levels? | Positive, negative, health, travel, cost |
| | What type of physical activity they would like? | Dance, weights, walking |
| **(ii)** | **care partner Previous and Current Physical Activity Levels** | |
| | What do you think of physical activity?<br>How does it make you feel? | Thoughts around it<br>Important part of life<br>Why/why not |
| | Are you physically active?<br>What factors influence your physical activity levels? | Me time |
| | What things would influence your decision to take part in an exercise class?<br>Motivators/barriers<br>Family/friends support<br>Can you tell me more about that?<br>How do you feel about that? | Interests? Friends?<br>Health benefits<br>Fun |
| | Has there been opportunities to participate in exercise groups since becoming a care partner of someone with dementia?<br>With the person you are caring for<br>On your own | |
| **(iv)** | **Preferences for Physical Activity Interventions** | |
| | What kind of exercise would you be interested in?<br>Type of exercise you would enjoy. | Dance? |

| | | |
|---|---|---|
| | If dance, what kind of dance would you enjoy?<br>Thoughts on a dance exercise class? | |
| | We are thinking of developing a dance class.<br><br>** PHOTOS OF PEOPLE DANCING**<br>I have brought a few photos with me today, of people dancing. I am hoping to hear what you think of these photos.<br>When you look at the photos how does it make you feel?<br>Does the photo remind you of anything?<br>What is your favourite photo and why? | |
| | Do you participate in exercise with the people living with dementia you care for?<br>If yes, what do you do?<br>If no, would you like to?<br>Do you enjoy it?<br>What would you like to do? | |
| | If there was an opportunity for a dance class for care partners and people living with dementia what do you think?<br>Good idea?<br>Would you have time to go | Why/why not<br>Be an interest<br>Adaptable different levels |
| | Class setting considerations:<br>What kind of setting should a class take place?<br>What time of the day would be best?<br>How would you get to the class?<br>What is an acceptable cost for a class? | Community hall/leisure centre<br>Just dementia and cg?<br>Mixed classes<br>Must be fun? Time? Place? |
| | Class structure considerations<br>How long a class would you like?<br>How often per week?<br>What level of intensity exercise would you like?<br>What kind of exercise class would you like to participate in? (one type of exercise, mixed exercise classes) | Easy/difficult/medium |
| | What things would be important to you for participating in exercise? | Must be fun<br>Type of exercise |
| | Is there anything you would like to ask me? | |

Supplementary File 3

| Reference number | Authors | Information supporting the intervention |
|---|---|---|
| 9 | Park and Cohen. 2019 | Long term exercise interventions can delay decreasing physical function. Improvements in physical function, including mobility and balance cane be seen in people living with dementia following exercise interventions. |
| 11 | Nimmons et al. 2023 | PAE reduces feelings of anxiety and depression in people living with dementia. |

| | | |
|---|---|---|
| 13 | Cunningham et al. 2020 | Physical inactivity is a risk factor for developing chronic conditions. Older adults who are physically active have a better QOL and improved cognitive functioning. |
| 17 | Lewis et al. 2020 | Dementia can lead to decreased physical function from impairment of their daily activities. |
| 18 | Fox et al. 2007 | Physical activity has positive impact on the mental health of older adults. |
| 19 | Boyle et al. 2014 | People with Alzheimer's dementia had low levels of physical activity and exercise compared to those with normal cognition. Physical activity is affiliated with high whole brain volume. |
| 29 | Van Alphen et al. 2016 | Physical activity benefits physical and cognitive function of people living with dementia. Physical inactivity can lead to comorbidities. Physical activity levels are low and decrease at a higher rate in people living with dementia an those without dementia. |
| 48 | Lambert et al. 2016 | care partners describe more negative health behaviours than people who are not care partners. |
| 49 | Lam et al. 2017 | Physical inactivity can cause further decline in physical functioning. |
| 50 | Mabire et al. 2019 | Dance as a psychosocial intervention has positive impacts on balance, gair, physical activities and QOL for people living with dementia. |
| 51 | Wu et al. 2021 | Dance improves memory, language and physical function in people with mild cognitive impairment. |
| 52 | Wang et al. 2022 | Aerobic and anaerobic exercise interventions have a positive impact on cognitive and physical function for people living with dementia. |
| | Orgeta et al. et al. 2014 | PA interventions can benefit caregivers through decreasing their stress, depression and burden. |
